# Molecular insights into a tetraspanin in the hydatid tapeworm *Echinococcus granulosus*

**DOI:** 10.1186/s13071-015-0926-y

**Published:** 2015-06-10

**Authors:** Dandan Hu, Xingju Song, Yue Xie, Xiuqin Zhong, Ning Wang, Yu Zheng, Xiaobin Gu, Tao Wang, Xuerong Peng, Guangyou Yang

**Affiliations:** Department of Parasitology, College of Veterinary Medicine, Sichuan Agricultural University, Ya’an, China; Department of Chemistry, College of Life and Basic Science, Sichuan Agricultural University, Ya’an, China

**Keywords:** *Echinococcus granulosus*, Tetraspanin, Immunolocalization, Immunogenicity, RNA interference

## Abstract

**Background:**

Cystic echinococcosis (hydatid disease), caused by the tapeworm *Echinococcus granulosus* (class Cestoda; family Taeniidae), is a neglected tropical disease that results in morbidity and mortality in millions of humans, as well as in huge economic losses in the livestock industry globally. Proteins from the tetraspanin family in parasites have recently become regarded as crucial molecules in interaction with hosts in parasitism and are therefore suitable for the development of vaccines and diagnostic agents. However, no information is available to date on *E. granulosus* tetraspanin.

**Methods:**

In this study, a uroplakin-I-like tetraspanin (Eg-TSP1) of *E. granulosus* was cloned and expressed in *E. coli*. The immunolocalization of Eg-TSP1 in different life stages of *E. granulosus* was determined using specific polyclonal antibody. The antibody and cytokine profiles of mice that immunized with recombinant Eg-TSP1 (rEg-TSP1) were measured for the immunogenicity analysis of this protein. Additionally, we use RNA interference method to explore the biological function of Eg-TSP1 in larva of *E. granulosus*.

**Results:**

Immunofluorescence analysis showed that endogenous Eg-TSP1 mainly localized in the tegument of larvae and adults. Significantly elevated levels of antibodies IgG1 and IgG2a and of cytokines IFN-γ and IL-12 were observed in the sera of mice after immunization with rEg-TSP1, suggesting a typical T helper (Th)1-mediated immune response elicited by rEg-TSP1. On further probing the role of Eg-TSP1 in *E. granulosus* by RNA interference, we found that a thinner tegmental distal cytoplasm was induced in protoscoleces treated with siRNA-132 compared to controls.

**Conclusions:**

This is the first report characterizing a tetraspanin from the tapeworm *E. granulosus*. Our results suggest that Eg-TSP1 is associated with biogenesis of the tegument and maintenance of structural integrity of *E. granulosus* and could therefore be a candidate intervention target for control of hydatid disease.

**Electronic supplementary material:**

The online version of this article (doi:10.1186/s13071-015-0926-y) contains supplementary material, which is available to authorized users.

## Background

Tetraspanins (TSPs) are a superfamily of plasma membrane associated proteins, also known as the trans-membrane-4-superfamily (TM4SF), which can be classified into four major subfamilies, including: the CD family (CD9, CD81, CD 151, *etc.*); the CD63 family (CD63, TSPAN31, *etc.*); the uroplakin family (UPK 1A/1B); and the retinal degeneration slow (RDS) family (RDS-ROM) [1]. These proteins usually consist of four conserved transmembrane domains, cytoplasmic tails at the N- and C-termini, a small extracellular loop, and a large extracellular loop (LEL) containing a Cys-Cys-Gly motif plus 2–6 additional cysteines [2]. These LELs have been proven to play a central role in interactions with several molecules, including the LELs of other tetraspanin, which is known as the “tetraspanin web” [3]. Tetraspanins participate in a broad spectrum of cellular activities, such as cell differentiation, adhesion, motility, aggregation, cell signaling and sperm-egg fusion [4–6], and are involved in many pathological processes, including cancer metastasis and infections caused by pathogenic organisms [7–10].

In spite of the importance of tetraspanins, only a small number have been studied to date [11]. In parasites, recent studies show that tetraspanins are associated with the development, maturation and stability of the tegument of trematodes, and are involved in the immune evasion of schistosome [12–16]. Of note, some members of the tetraspanin family have been targeted as candidate vaccines against schistosomiasis [17–20], clonorchiasis [21], alveolar echinococcosis [22–25], and filariasis [26]. For example, inoculation with the LELs of two tetraspanins of *S. mansoni* (Sm-TSP-1 and Sm-TSP-2) was confirmed to significantly decrease the adult worm burdens and liver egg burdens in experimentally infected mice [20]. Moreover, a *Taenia solium* tetraspanin (T24) was found to be a potential diagnostic candidate, which exhibited excellent sensitivity and specificity in detecting cases of cysticercosis with two or more viable cysts [27].

*Echinococcus granulosus* is a causative pathogen of human and domestic animal hydatid disease, infects ~3 million people globally and causes annual losses of US$2 billion in livestock [28, 29]. *E. granulosus* has recently been reported to have more tetraspanin genes (30) than *Caenorhabditis elegans* (20) and other parasitic nematodes (≤20) [1, 30]. However, there are no further reports about the potential roles of tetraspanins in *E. granulosus* to date.

In this study, we characterized a tetraspanin, Eg-TSP1, from *E. granulosus* and investigated its locations in different life cycle stages. The immune mechanism in mice immunized with the recombinant LEL region of Eg-TSP1 (rEg-TSP1) was also evaluated by measuring the levels of IgG and IgG subclasses (IgG1 and IgG2a), and of cytokines interleukin (IL)-4, IL-10, IL-12 and interferon (IFN-γ). In addition, to further explore the potential role of Eg-TSP1 in *E. granulosus*, RNA interference (RNAi) was employed to suppress the endogenous gene expression in its larvae (protoscoleces, PSCs) *in vitro*. To our knowledge, this is the first report that focuses on the functional genomics of *E. granulosus* by means of RNAi, and the results will provide the foundation for future research into prevention and control of hydatid disease.

## Methods

### Ethics statement

This study was reviewed and approved by the National Institute of Animal Health Animal Care and Use Committee at Sichuan Agricultural University (Ya’an, China; approval no. 2013–028). All animal experiments were conducted in accordance with the Regulations for the Administration of Affairs Concerning Experimental Animals (approved by the State Council of the People’s Republic of China).

### Animals

Six- to eight-week-old female specific pathogen free (SPF) ICR mice were purchased from the Laboratory Animal Center of Sichuan University (Chengdu, China). A 15-week-old male New Zealand white rabbit and a 5-month-old dog of mixed breed were obtained from the Laboratory Animal Center of Sichuan Agricultural University.

### Parasites

Cysts of *E. granulosus* were collected from an infected sheep at a local slaughterhouse in Xining, Qinghai Province, China. PSCs and cyst wall (including laminated layer and germinal layer) were separated under sterile conditions and washed in phosphate-buffered saline (PBS). Fresh PSCs were diluted to a concentration of 2,000 mL^−1^ and then cultured in complete RPMI 1640 medium (Hyclone) containing 10 % fetal calf serum (Hyclone), 100 U.mL^−1^ penicillin G and 100 μg.mL^−1^ streptomycin (Sigma) at 37 °C in an atmosphere containing 5 % CO_2_. Adult worms were collected from a dog 35 days post-infection with 20,000 PSCs.

### Bioinformatic analysis

Genome-wide tetraspanins of *E. granulosus* were download from GeneDB (http://www.genedb.org/) according to the summary by Tsai *et al.* [30], and were aligned and employed for phylogenetic analysis by MEGA software (version 5.05). Full length *E. granulosus* TSP1 nucleotide sequence [GenBank: FJ384717] was downloaded from GeneDB [31] based on sequence homology with *E. multilocularis* TSP1 [GenBank: FJ384717]. Open Reading Frame Finder (http://www.ncbi.nlm.nih.gov/gorf/gorf.html) and MEGA software were used to analyze the open reading frame (ORF) and deduce the amino acid sequence. To find other tetraspanins, the amino acid sequence of Eg-TSP1 was used for BLAST against the National Center for Biotechnology Information (NCBI) database. Multiple sequence alignment was performed using the DNAStar program (Madison WI, USA), and the aligned full-length amino acid sequences were employed to build a phylogenetic tree using Mrbayes version 3.1.2 [32]. The hypervariable region of the tetraspanins was compared using the Clustal W2 online service. The molecular weight and transmembrane regions were predicted using ProtParam (http://web.expasy.org/protparam/) and DAS Transmembrane Prediction server (http://www.sbc.su.se/~miklos/DAS/), respectively.

### Cloning, expression and purification of the LEL region of Eg-TSP1

The total RNA of PSCs was isolated using Trizol reagent (Invitrogen, Carlsbad, CA, USA) and then reverse transcribed into cDNA using the ThermoScript™ RT-PCR System for First-strand cDNA Synthesis Kit (Invitrogen). The nucleotide sequence encoding the LEL domain of Eg-TSP1 was amplified from cDNA using a sense primer (5′-CGC*GGATCC*CCTGATAACCTAAACAAAGC-3′) containing a *BamH*I site (underlined) and an antisense primer (5′-CCC*AAGCTT*GAGGGTTTTGTTCTCTGCCAA-3′) containing a *Hind*III site (underlined). The PCR products were ligated into the pET32a(+) plasmid (Novagen, Madison, WI, USA). The recombinant plasmid with correct sequence was transformed into *Escherichia coli* BL21 (DE3) cells (Invitrogen) and subsequently grown at 37 °C in Luria-Bertani broth containing 50 μg.mL^−1^ ampicillin. When the OD_600nm_ value of bacteria reached 0.6, 1 mM isopropyl-β-D-thiogalactopyranoside (IPTG) was added for 5 h induction at 37 °C. The recombinant protein was harvested from *E. coli* lysates, followed by purification with a Ni^2+^ affinity column (Bio-Rad, USA) under denaturing conditions in 8 M urea. The purified protein was refolded by dialysis against PBS and concentrated using an Amicon Ultra-15 10,000 MWCO centrifugal filters (Millipore, USA), and then analyzed by SDS-PAGE.

### Sera

Mouse anti-PSC sera was produced as described previously [33] and used for immunoblot analysis. Briefly, each mouse was inoculated intraperitoneally with 200 μL of a suspension containing 2,000 viable protoscoleces, and then serum was acquired 9 month later. For immunolocalization, a rabbit was immunized with a subcutaneous injection of 50 μg.mL^−1^ of rEg-TSP1 purified as described above mixed with Freund’s complete adjuvant (FCA; Sigma, St. Louis, MO, USA), followed by two booster immunizations (2 weeks apart) using the same route and dose in Freund’s incomplete adjuvant (FIA; Sigma). The rabbit was bled 2 weeks after the second booster immunization. The polyclonal antisera were collected, purified by Protein A affinity chromatography (Bio-Rad, USA), and stored at −80 °C until use.

### Immunoblotting and immunolocalization

For immunoblotting, rEg-TSP1 was run on 12 % SDS-PAGE and then transferred onto a nitrocellulose membrane. After blocking, the membrane was incubated with mouse anti-PSC sera (1:200), followed by goat anti-mouse IgG (H+L) HRP conjugate (1:2,000) (Bio-Rad). Nitroblue tetrazolium and 5-bromo-4-chloro-3-indolyl phosphate (NBT/BCIP; USA, Cleveland, OH) were used as substrates to develop the color reaction. The serum of non-infected mice was used as a control. The specificity and sensitivity of rabbit anti-rEg-TSP1 IgG were determined by western blotting against crude extracts from PSCs and rEg-TSP1 as described above.

To determine the location of Eg-TSP1, fresh PSCs, cyst wall (including the laminated layer and germinal layer), and adult worms were fixed in 4 % paraformaldehyde-phosphate buffer for 36 h and then embedded in paraffin after dehydration. Sections were deparaffinated and rehydrated, and subsequently treated with 0.01 M citrate buffer (pH 6.0) in a microwave at 700 W for 10 min for antigen exposure, followed by blocking with 3 % H_2_O_2_. Then these sections were washed and incubated with purified IgG fractions (diluted 1:200 in PBS) at 4 °C overnight. The sections were washed and incubated with fluorescein isothiocyanate (FITC)-conjugated goat anti-rabbit IgG (H+L) (Bethyl Laboratories) at 37 °C for 1 h in darkness. After four washes with PBS, sections were examined under a fluorescence microscope (Nikon, Japan). Antibody from pre-immunized rabbit was used as negative control.

### Immunization experiment

In the immunized group, eight mice were subcutaneously injected with rEg-TSP1 (25 μg per animal) mixed with FCA (Sigma), followed by three boosters separately performed on days 14, 28 and 42 using the same route and dose in FIA (Sigma). In the control group, eight mice were treated in the same way as immunized group with the replacement of rEg-TSP1 into PBS. Sera were collected for antibody analysis at days 0 (pre-immunization), 14, 28, 42 and 56. Two weeks after the final immunization, all mice were sacrificed and the spleens were removed aseptically for cytokine assays.

### Antibody assays

The levels of antibodies including IgG, IgM, IgE, and IgG subclasses against rEg-TSP1 were measured by ELISA. In brief, 96-well plates were coated with rEg-TSP1 (2 μg.mL^−1^) using 100 μL reaction volume at 4 °C for 14–16 h. After three washes, the plates were blocked with PBS containing 5 % skimmed milk for 1 h at 37 °C. The mouse antisera (1: 200) was subsequently added and incubated for 1 h at 37 °C, followed by HRP-conjugated goat anti-mouse IgG (1:500), IgM (1:5,000), IgE (1:5,000), or IgG subclass (IgG1, IgG2a; 1:5,000) antibodies (Bethyl Laboratories). Antibody binding was detected at 37 °C with 100 μL of 3,3′,5,5′-tetramethylbenzidine (TMB; Tiangen, China) and the reaction was stopped by adding 100 μL of 2 M H_2_SO_4_. ELISA OD values were measured at 450 nm using a microplate reader (Thermo Scientific, USA). All samples were run in triplicate.

### Cytokine assays

The cytokine profiles (IL-4, IL-10, IL-12 and IFN-γ) of immunized mice were measured using quantitative PCR (qPCR). The amplification primer pairs are shown in Additional file 1. For amplification, total RNA was isolated from mouse spleens, and first-strand cDNA was synthesized as described above. All real-time PCR amplifications were carried out in a MiniOpticon thermal cycler (Bio-Rad) in a total volume of 12.5 μL, containing 1 μL of cDNA, 6.25 μL of SYBR® Premix Ex Taq™ II (Takara, China), 0.5 μM of each primer, and nuclease free water. The amplification steps included initial denaturation at 95 °C for 30s, followed by 40 cycles of amplification, each including denaturation at 95 °C for 30 s, annealing at 55.0–59.7 °C for 30 s, and extension at 72 °C for 30 s. The reactions were performed in triplicate, with negative controls. Dissociation curves were analyzed for each sample to confirm the specific amplification. The relative gene expression levels were calculated using the comparative 2^-ΔΔCt^ method [34] and normalized with the hypoxanthine phosphoribosyl transferase (HPRT) gene.

### RNAi and qPCR

To further investigate the role of Eg-TSP1 protein in the larval stage of *E. granulosus*, we knocked down the expression level using small interfering RNA (siRNA). Three specific siRNAs were designed to target different CDS regions of Eg-TSP1 (Additional file 2) and were chemically synthesized by GenePharma Co., Ltd. (Shanghai, China); and an irrelevant siRNA, that was non-specific to any sequence of *E. granulosus*, was also synthesized as negative control. For delivery of siRNAs, PSCs were treated with 10 μL of transfection reagent (Engreen Biosystem, China) plus siRNA at a final concentration of 200 μM in 2.5 mL culture medium at days 0 and 2. Medium was changed daily.

To confirm the knockdown effect of RNAi in PSCs, the transcription level of Eg-TSP1 was determined by qPCR. Approximately 2,000 PSCs were collected for total RNA isolation and cDNA synthesis 24 h after the final siRNA delivery. The qPCR primer pairs of Eg-TSP1 and elongation factor 1 alpha (EF1α, an optimized housekeeping gene in *Echinococcus* spp. [35]) were given in Additional file 1. All amplifications were performed as described above, except for the annealing temperatures: 53.2 °C for Eg-TSP1 and 57.8 °C for EF1α. qPCR was conducted in triplicate and Eg-TSP1 transcript levels were calculated by 2^-ΔΔCt^ analysis.

### TEM analysis

For transmission electron microscope (TEM) analysis, PSCs were treated with Eg-TSP1-specific and irrelevant siRNAs for 4 days at 37 °C in an atmosphere containing 5 % CO_2_. Then, PSCs were fixed with 3 % glutaraldehyde in 0.1 M PBS for 48 h at 4 °C, followed by post-fixing using 2 % OsO_4_. Fixed PSCs were dehydrated in graded acetones (50–100 %) and embedded in Epon Resin (ProSciTech, Australia). Sections (60 nm) were mounted onto copper grids, stained with uranyl acetate and lead citrate, and observed using a TEM (Rigaku, Japan) operated at 75 kV. This experiment was performed in triplicate.

### Statistical analysis

All data are presented as mean ± SD. Statistical analyses were performed with Student’s *t*-test for comparison between groups using the software package GraphPad Prism (www.graphpad.com).

## Results

### General characteristics of Eg-TSP1

**Fig. 1:**
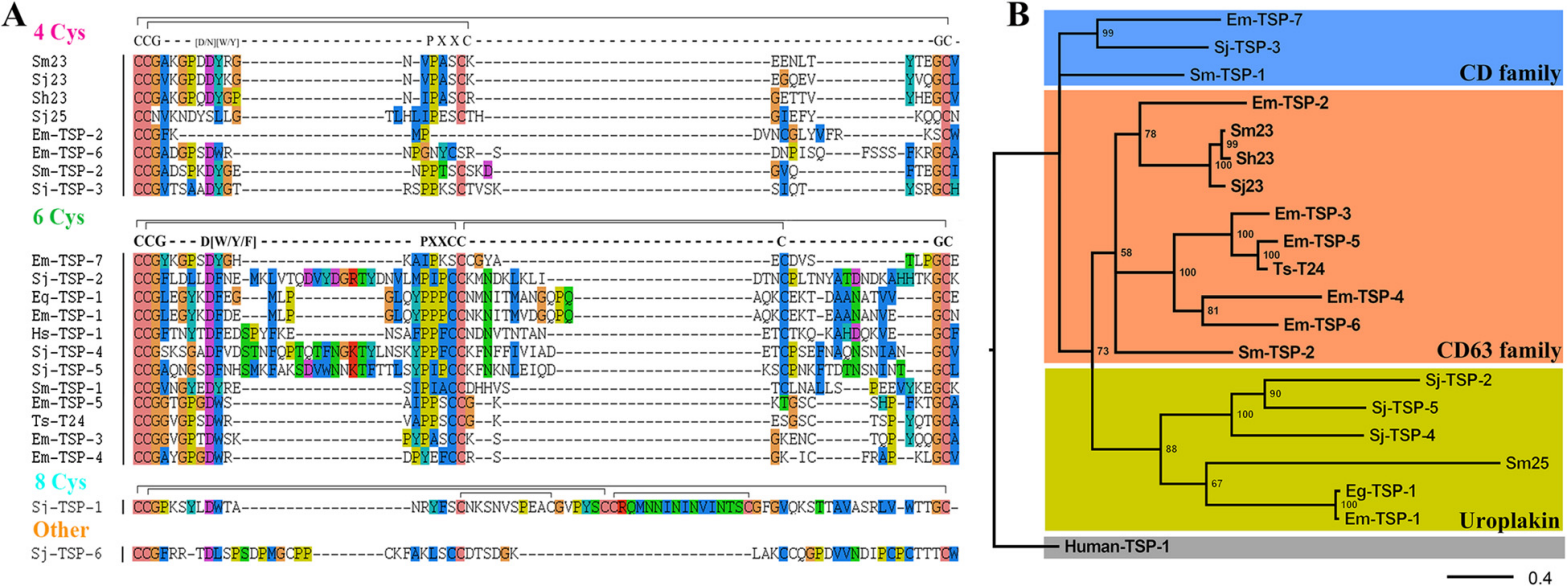
Phylogenetic analysis and the cysteine pattern in Eg-TSP1. **a** The hypervariable region of tetraspanins was aligned and they were grouped according to their cysteine patterns. The conserved cysteine-motif is summarized and the canonical topologies of disulfide bonds are shown above the alignment. **b** A phylogenetic tree was constructed based on the amino acid sequences of parasite and human tetraspanins using the Bayesian method. Three major monophyletic subfamilies were detected, including the CD family, the CD63 family, and the Uroplakin family. Eg, *E. granulosus*; Em, *E. multilocularis*; Sm, *S. mansoni*; Sj, *S. japonicum*; Sh, *S. haematobium*; Ts, *T. solium*; Hs, *Homo species*. GenBank accession numbers: Em-TSP 1–7 [FJ384717, FJ384718, FJ384719, FJ384720, FJ384721, FJ384722 and FJ384716, respectively]; Sj-TSP 1–6 [AAW26928, AAW24822, AAW24863, AAP05954, AAW27174 and AAW26326, respectively]; Sm-TSP-1 [AF521093]; Sm-TSP-2 [AF521091]; Sm23 [M34453]; Sj23 [M63706]; Sh23 [U23771]; Sm25 [AF028730]; Sj25 [U77941]; Ts-T24 [AY211879] and Human-TSP1 [NP_005718.2]

The full-length 792 bp CDS of Eg-TSP1 encoded 263 residues including the four typical transmembrane regions (9–32 aa; 70–92 aa; 100–123 aa; and 236–255 aa), the small extracellular loop (33–69 aa), and the crucial LEL located in amino acids 124–235. Alignment of the hypervariable regions in the LEL of parasite and human tetraspanins showed different cysteine distributions (Fig. 1a). Most of the sequences contained four or six cysteine residues, while only Sj-TSP1 and Sj-TSP6 had ≥8 cysteine residues. Eg-TSP1 had a six-cysteine motif: CCG…DF…PXXCC…C…GC. Notably, the residues CCG, the first C of PXXCC, and GC in this motif were highly conserved.

On searching against the conserved domain database of NCBI using the amino acid sequence of Eg-TSP1, we found that there was high homology between Eg-TSP1 and uroplakin-I-like family (Accession: cd03156; E-value: 2.64e-06). Additionally, our Bayesian analysis (Fig. 1b) showed three distinct branches, including members of the CD family, uroplakins, and the CD63 family. *E. granulosus* Eg-TSP1 belonged to the same clade as Sj-TSP2/4/5, Em-TSP1 and Sm25. Further, all tetraspanins of *E. granulosus* could also be classified into three parts, and Eg-TSP1 (GeneDB ID: EgrG_000355800.1) together with a single paralogue (GeneDB ID: EgrG_000355900.1) (equal to LophDB Cluster ID: EGC00290, http://www.nematodes.org/NeglectedGenomes/Lopho/; and showed absence of ESTs in transcriptome data of PSCs [36]) consist of a independent branch belonged to uroplakin family (see Additional file 3).

### Expression, purification and recognition of rEg-TSP1

**Fig. 2:**
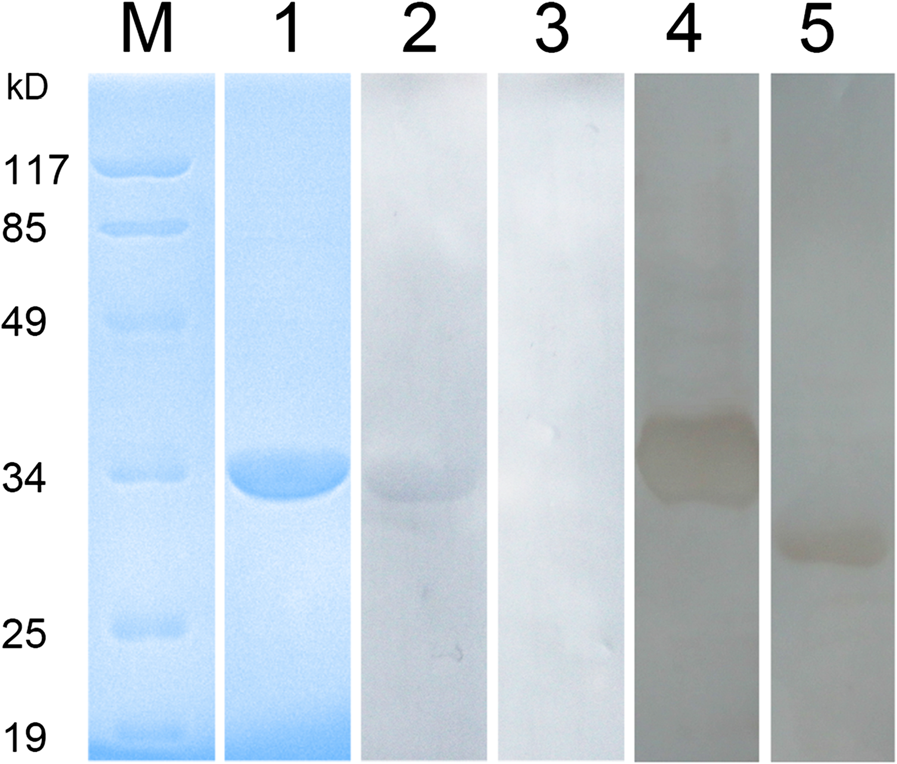
Immune recognition of recombinant and native Eg-TSP1 by Western blot. Lane 1, SDS-PAGE of purified rEg-TSP1 after dialysis and concentration; Lane 2 and lane 3, immune recognition of rEg-TSP1 using sera from *E. granulosus* infected mice (Lane 2) and healthy control (Lane 3); Lane 4 and lane 5, western blot analysis using recombinant Eg-TSP1 (Lane 4) and crude extracts of protoscolexes (Lane 5) against the purified rabbit anti-rEg-TSP1 IgG. A single band was detected in each lane with molecular weight of ~32 kDa for recombinant Eg-TSP1 (partial sequence plus ~20 kDa fusion peptide) and ~29 kDa for native Eg-TSP1, respectively

A 330 bp fragment encoding the LEL of Eg-TSP1 was amplified from PSCs and this fragment was expressed in *E. coli* as a His_6_-tagged fusion protein with an expected size of ~32 kDa (Fig. 2). Minus a ~20 kDa epitope tag fusion peptide, rEg-TSP1 had an approximate molecular mass of 12 kDa, which was similar to the MW of the LEL predicted *in silico*. Immunoblotting using sera from mice experimentally infected with *E. granulosus* showed a single ~32 kDa band (Fig. 2), indicating that rEg-TSP1 can be recognized by the mouse specific IgG against *E. granulosus*.

### Localization of Eg-TSP1 in different life cycle stages of *E. granulosus*

**Fig. 3:**
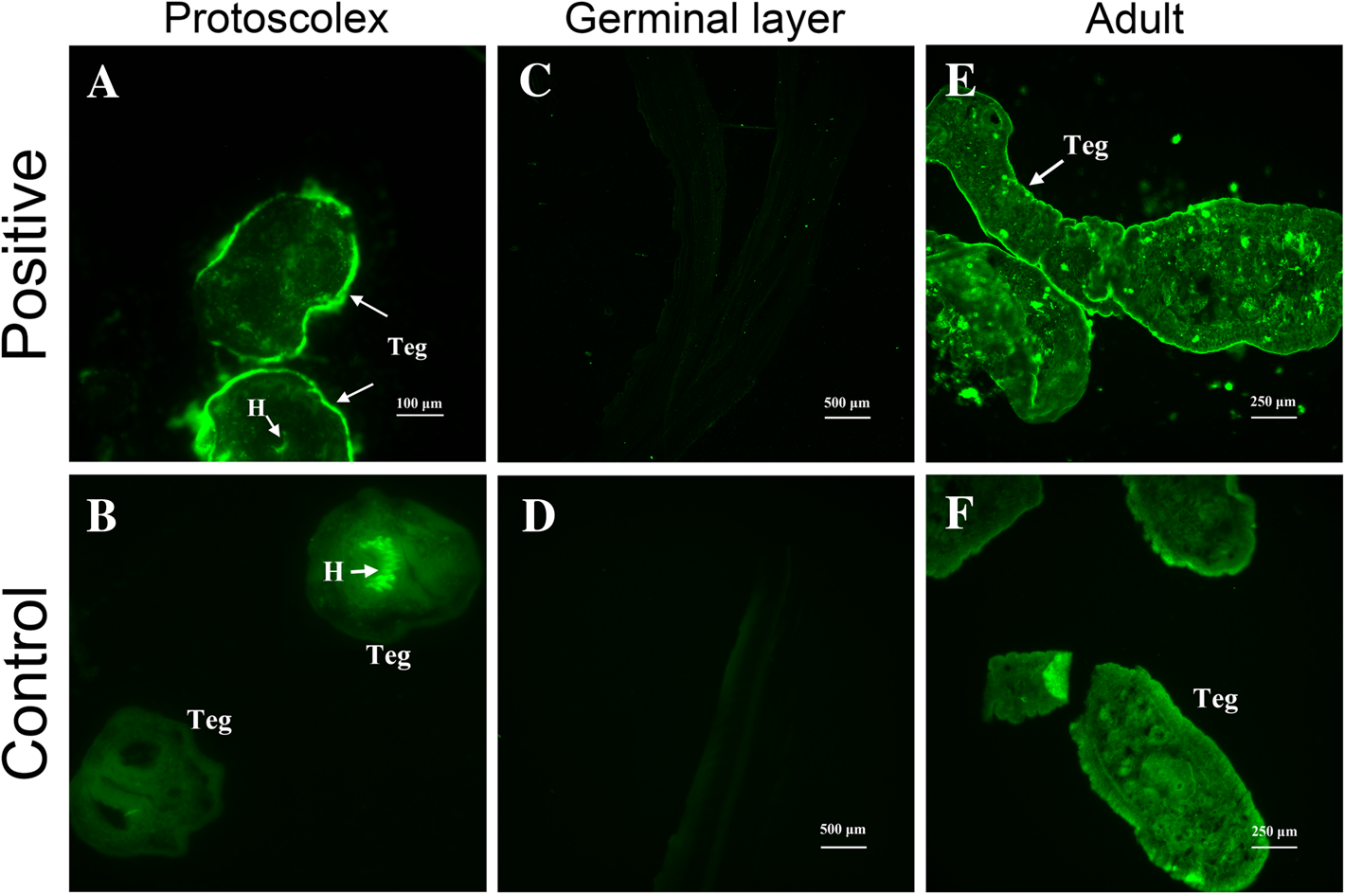
Immunolocalization of Eg-TSP1 in the larval and adult stages of *E. granulosus*. Eg-TSP1 in the protoscolexes (**a, b**), germinal layer (**c, d**) and adult (**e, f**) was immunofluorescently labeled using specific anti-rEg-TSP1 IgG (**a, c** and **e**), or control pre-immune serum (**b, d** and **f**), followed by FITC-conjugated anti-rabbit IgG. Fluorescence-labeled regions are marked with arrows. Teg: tegument; H: hooks

The localizations of native Eg-TSP1 in PSCs (Fig. 3a, b), cyst wall germinal layer (Fig. 3c, d), and adult worms (Fig. 3e, f) were determined by immunofluorescence using specific polyclonal antibodies against rEg-TSP1 (Fig. 2). The Eg-TSP1 was located only in the tegument and invaginated sucker of PSCs. No signals were detected in the germinal layer of cyst walls, or in the non-cell structural laminated layer (data not shown).

### Antibody profiles

**Fig. 4:**
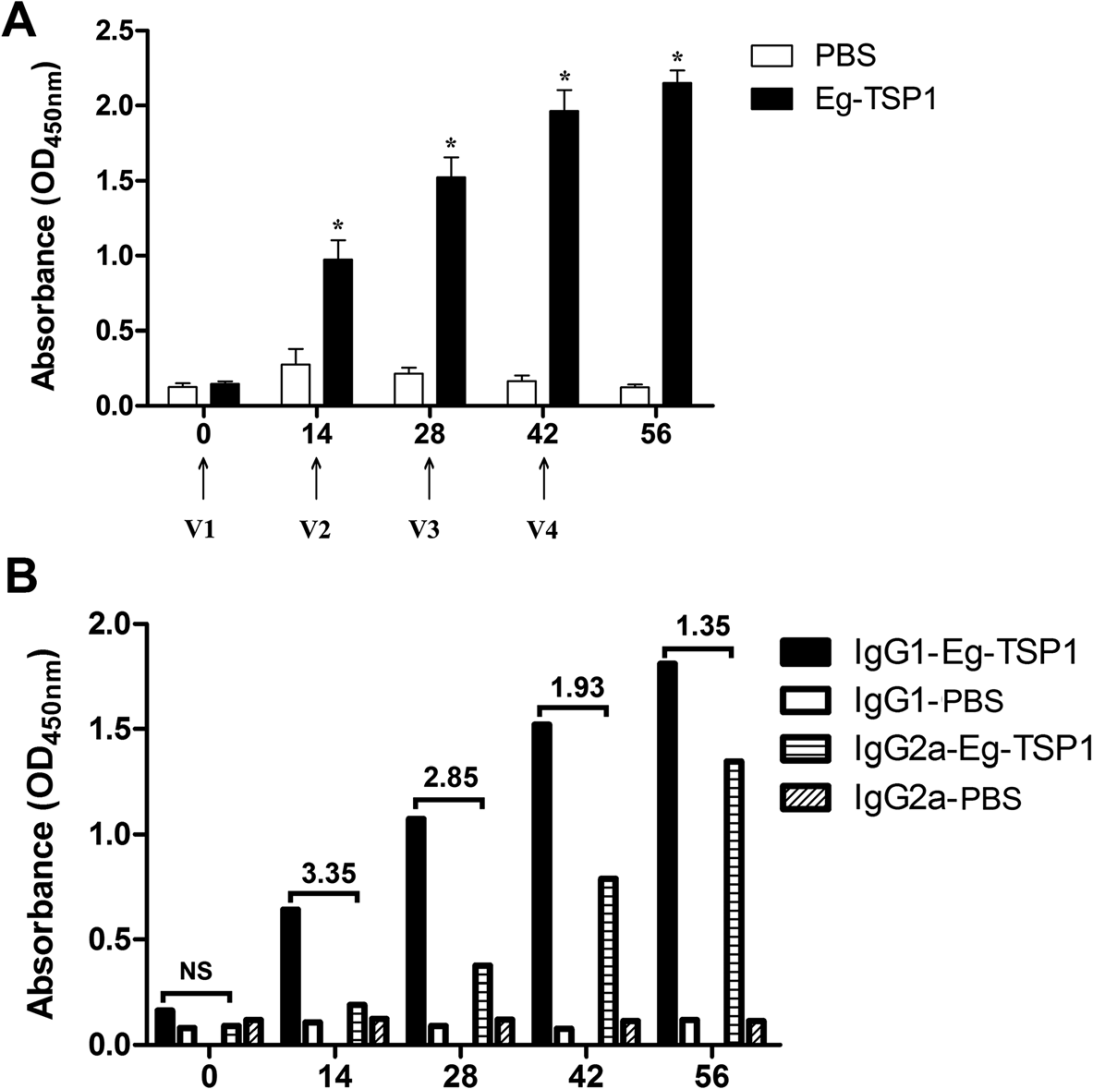
Serum antibody profiles induced by rEg-TSP1 in mice. Eight mice per group were subcutaneously vaccinated four times with rEg-TSP1 or PBS mixed with FCA/FIA at 14 day intervals and serum samples were collected via the tail vein for ELISA tests after each vaccination. **a** Serum IgG levels of mice vaccinated with rEg-TSP1. Vaccination times are marked with arrows. **b** Serum IgG subclass levels of mice vaccinated with rEg-TSP1. The ratio of IgG1/IgG2a were given above the bars. Asterisks indicate statistically significant differences between the rEg-TSP1 and control groups (*P* < 0.0001). NS represent not significant. Error bars represent SD

To evaluate the levels of specific IgG, IgM, IgE and IgG subclass (including IgG1 and IgG2a) antibodies to rEg-TSP1, sera from eight immunized mice were measured at 0, 14, 28, 42 and 56 days after first vaccination. Compared with the PBS control group, significant IgG responses were observed after the first immunization in the group immunized with rEg-TSP1, and the peak was detected 56 days post-vaccination (Fig. 4a). We did not find any significant differences in rEg-TSP1-specific IgM and IgE levels at any time points we studied (data not shown). To evaluate the IgG isotype profiles induced by rEg-TSP1, specific IgG1 and IgG2a antibody levels were measured. Both IgG1 and IgG2a levels were significantly increased at 14, 28, 42 and 56 days post-immunization compared to the controls. The IgG2a level was lower than the IgG1 level at all time points but, interestingly, the IgG1/IgG2a ratio declined at days 28, 42, and 56 due to a fast increase in IgG2a (Fig. 4b), suggesting a Th1-type response tendency.

### Cytokine response induced by rEg-TSP1

**Fig. 5:**
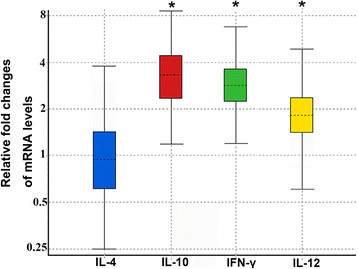
Relative expression levels of cytokines determined in mice post-immunization with rEg-TSP1 or PBS. Spleens were isolated at day 56 from mice subcutaneously immunized with rEg-TSP1 or PBS. Total RNAs were extracted and then cDNAs were synthesized for qRT-PCR analysis. *P < 0.001. Boxes represent the interquartile range, *i.e.* the middle 50 %, of observations. The dotted line represents the median gene expression. Whiskers represent the minimum and maximum observations

Mouse spleens were used for the detection of cytokine expression profiles 56 days after vaccination with rEg-TSP1 or PBS. From the qPCR data (Fig. 5), we clearly found that the levels of IFN-γ and IL-12 (cytokines of Th1 cell response) were highly elevated, while IL-4 (a typical cytokine of Th2 immune response) showed no difference compared to the control group (*P* > 0.05). These observations suggested a Th1 immune response induced by rEg-TSP1 vaccination. However, a high level of IL-10 (a Th2 cytokine) was also detected (Fig. 5).

### SiRNA mediated knockdown of Eg-TSP1 transcript

**Fig. 6:**
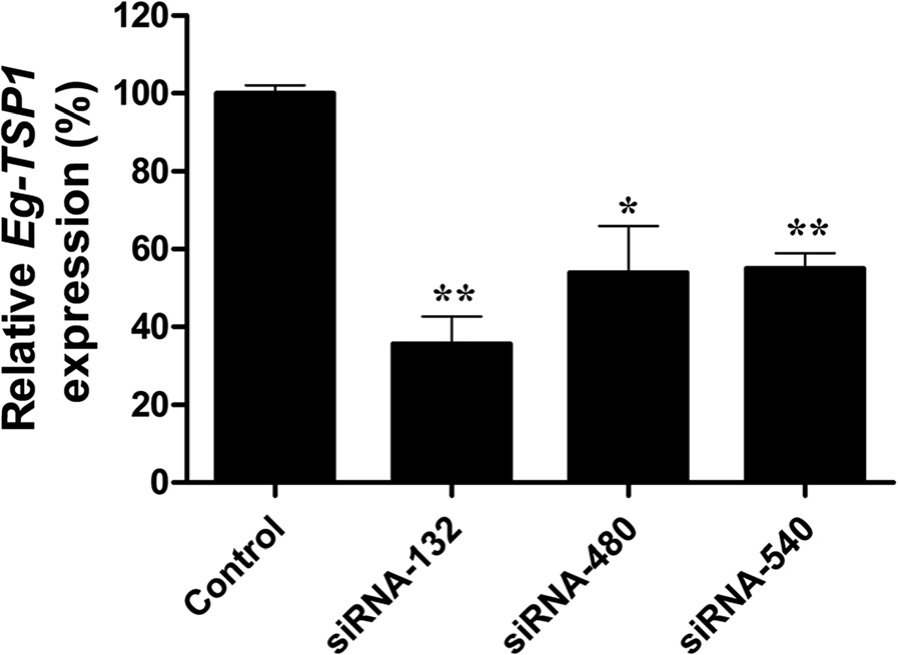
Suppression of Eg-TSP1 transcripts in *E. granulosus* protoscoleces using different siRNAs. siRNAs were transferred twice to *E. granulosus* protoscoleces by soaking. An irrelevant siRNA was used as the control and three Eg-TSP1-specific siRNAs (siRNA-132, −480 and −540, respectively) were the experimental samples. Eg-TSP1 transcript levels were measured by qRT-PCR and normalized using Eg-EF1α transcripts. Data are presented as the mean ± SD of triplicate experiments. Statistically significant differences between the control group and the Eg-TSP1-specific siRNA groups were determined by Student’s *t*-test (* *P* < 0.01; ** *P* < 0.0001)

SiRNAs (siRNA-132, −480 and −540) were introduced into PSCs to mediate gene knockdown using two transfections, and caused 64.30 % (*P* = 0.0001), 46.03 % (*P* = 0.0028) and 44.93 % (*P* < 0.0001) decreases in mRNA transcripts compared to the negative control group, respectively (Fig. 6). The results showed that siRNA-132 was the most efficient siRNA and that the effect of inhibition by siRNA was sequence-dependent.

### Structural changes in parasites caused by RNAi

**Fig. 7:**
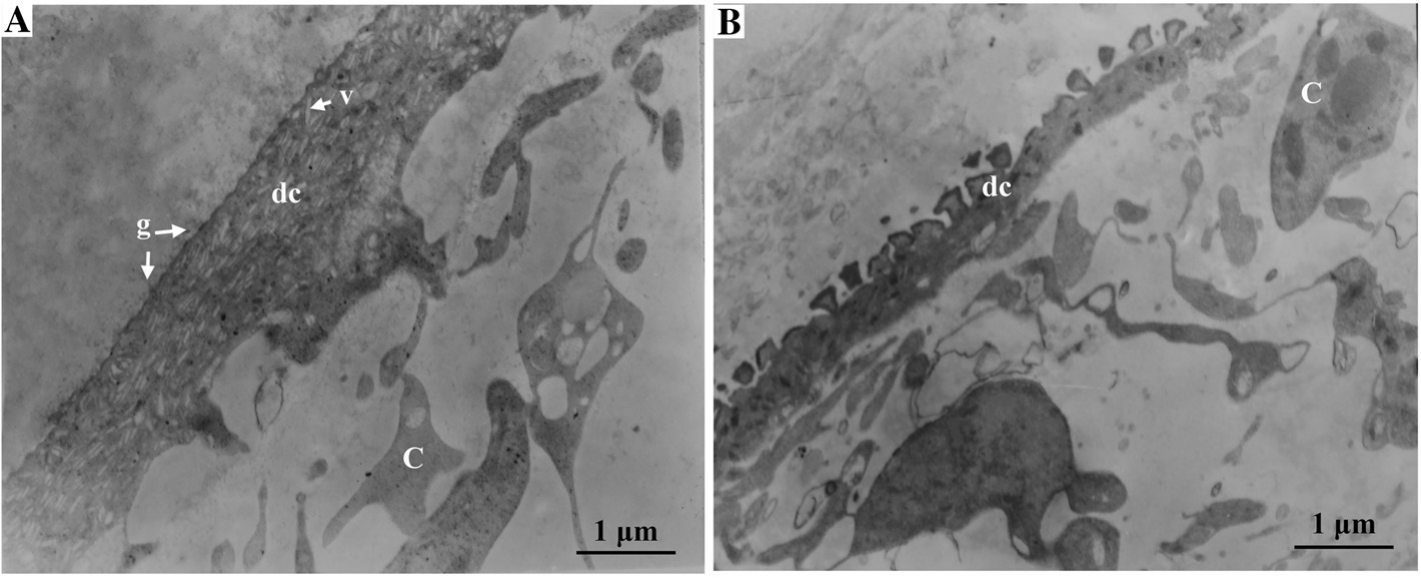
Ultrastructure of the tegument of *E. granulosus* protoscoleces treated with Eg-TSP1 siRNA. g: glycocalyx; v: vesicle; dc: distal cytoplasm; C: parenchyma cell. **a** The distal cytoplasm in the somal regions of protoscoleces treated with siRNA-132 is observed to be thinner than that in (**b**) protoscoleces treated with irrelevant siRNA. Analysis was by transmission electron microscopy (magnifications 10000×)

To investigate the effect of inhibition of Eg-TSP1 expression in *E. granulosus in vitro*, PSCs that were treated with the most efficient siRNA (siRNA-132) or irrelevant siRNA were observed by TEM. The tegument structure of PSCs that were exposed to Eg-TSP1-specific siRNA was significantly different from that of the control group soaked in the irrelevant siRNA (Fig. 7). The tegumental distal cytoplasm of PSCs treated with siRNA-132 was much thinner than that of the controls, with less vesicles inside.

## Discussion

Tetraspanins are transmembrane proteins widely distributed in eukaryotic organisms [37], involved in many cell biological processes [38, 39], and that interact with immune molecules from hosts [40], especially specific molecules such as the major histocompatibility complex (MHC) II [41, 42]. These interactions tend to downregulate host immune responses so that parasites can mask their nonself status to escape host detection and successfully establish infection in the host. Thus, targeting tetraspanins via various methods such as monoclonal antibodies, soluble large-loop proteins or RNAi technology, should be therapeutically valuable [43]. Recent studies on parasite tetraspanins as vaccine candidates have made great progress and given this protein family an important status. However, there were no experimental data about tetraspanins in the zoonotic platyhelminth pathogen, *E. granulosus*. In this study, we first characterized a uroplakin-I-like tetraspanin on the surface of *E. granulosus* and found that it plays a crucial role in the tegument formation of this parasite.

In this study, the amino acid sequence of Eg-TSP1 showed the classical “CCG” pattern of tetraspanins and shared high similarity with uroplakin-I-like Sj-TSP2/4/5 [44], suggesting that Eg-TSP1 is another uroplakin-I-like tetraspanin in platyhelminths and enriches the spectrum of this protein family. Uroplakins Ia and Ib (special tetraspanins) specifically bind with their associated (non-tetraspanin) uroplakins II and IIIa, and together form two dimensional crystals of 16 nm particles, known as urothelial plaques, which cover almost the entire apical surface of the mammalian bladder urothelium, modulating the urothelial permeability barrier [45, 46], and are involved in urinary tract infection [47]. However, *E. granulosus* is a tapeworm without a bladder and urinary tract; thus, we can speculate that mammals and cestodes belong to distinct animal lineages and their tetraspanins diverged with the divergence of the groups, coming to be expressed in different tissues and played different roles [46]. Hence, further exploration of the structure and function of Eg-TSP1 was required.

We confirmed the localization of Eg-TSP1 in the larval and adult stages of *E. granulosus* by immunofluorescence, and the results showed that Eg-TSP1 was mostly present in the tegument. As mentioned previously, *E. granulosus* is a cestode devoid of a gut, and the tegument with microvilli or microtriches constitutes the principal site of absorption of nutrients and elimination of waste materials, similar to the gut of animals turned inside out [48]. Thus, many proteins located on the tegument of *E. granulosus* should be contributed to parasite nutrition. Given the primary function of uroplakin in mammals is to regulate cell permeability, we can speculate that Eg-TSP1 may have a role in managing flow of materials in and out of the parasite. However, in this study, we have not excluded the possibility that Eg-TSP1 is expressed in the ducts of the excretory system of the parasites, so further detailed analysis should be done. We did not detect the expression of Eg-TSP1 on the germinal layer of cyst walls, in contrast to its homologue TSP1 in *E. multilocularis* [23]. However, this result was supported by transcriptome data for *E. granulosus* [36], where reads of Eg-TSP1 (LophDB Cluster ID: EGC00446; http://www.nematodes.org/NeglectedGenomes/Lopho/) in the cyst wall were also not detectable. Considering that rEg-TSP1 could be recognized by the mouse-anti-PSCs serum, it seems like that Eg-TSP1 interact with host immune system in the context of secondary infection, when *E. granulosus* cysts were broken.

Dang *et al.* [23] evaluated protective effects of seven *E. multilocularis* tetraspanins on the murine-alveolar echinococcosis model; the recombinant LEL of Em-TSP1 induced the highest cyst lesion reduction rate (87.9 %). However, a lower cyst lesion reduction rate was detected when using another immunization strategy [24]. Here, we measured the levels of antibodies in mice immunized with rEg-TSP1 and found an increasing rEg-TSP1-specific IgG level after the second immunization. Moreover, analysis of the IgG1/IgG2a ratio showed a tendency towards a protective Th1 type of immune response induced by rEg-TSP1. This was confirmed by the significantly upregulated levels of splenic IL-12 and IFN-γ and an unchanged level of IL-4 in the cytokine profile. Of note, IL-12 is a key cytokine in the development of Th1 immunity and can facilitate Th1 response by enhancing the secretion of IFN-γ by T cells and NK cells, but plays an antagonist role in the production of IL-4. Furthermore, IFN-γ is important in protective immune response against parasite infections since it enhances the secretion of IgG2a from B lymphocytes [49], thus resulting in a decrease of the IgG1/IgG2a ratio and triggering the Th1 response. In the case of cystic echinococcosis, IFN-γ was involved in activation of macrophages for production of nitric oxide synthase that can induce host defense against *E. granulosus* [50]. In addition, a high level of IL-10 is a hallmark of chronic *E. granulosus* infection [51]. Here, we detected high-level IL-10 production in mice after immunization with rEg-TSP1, so we hypothesize that this cytokine might be involved in the regulation of Th2 response and the prevention of a highly polarized Th1 response in which hosts might tolerate the parasite infection [52, 53]. Further confirmation of the protective effect of rEg-TSP1 may require a challenge experiment in the mouse model.

RNAi has been widely used to knockdown genes in nematodes and trematodes (reviewed by Maule *et al.* [54] and Geldhof *et al.* [55], respectively), but there are few reports of RNAi in cestodes except *Moniezia expansa* [56] and *E. multilocularis* [57, 58]. Mizukami *et al.* [57] successfully knocked down *E. multilocularis* PSC *14-3-3* and *elp* gene expressions to 21.8 % and 35.5 %, respectively, and demonstrated that the gene knockdown happened only on siRNA delivery by electroporation and not by soaking in siRNA with transfection reagent. However, in this study, PSCs of *E. granulosus* soaked with Eg-TSP1-specific siRNA in transfection reagent showed a statistically significant decrease in Eg-TSP1 gene expression that led to malformation of the worm tegument, compared to samples treated with an irrelevant siRNA. These results suggested a role for Eg-TSP1 in biogenesis of the tegumental distal cytoplasm and maintenance of structural integrity. Importantly, they also demonstrate that soaking is a viable approach, with fewer cell lesions than electroporation, when transferring siRNA to PSCs of *E. granulosus*. The use of RNAi in functional genomic analysis of *E. granulosus* will facilitate the decoding of functions of many important genes and contribute to prevention and control of human and domestic animal hydatid disease [59].

## Conclusion

In conclusion, more and more attention is being paid to the proteins of the tetraspanin superfamily because of their wide distribution and crucial biological roles in various organisms. In this study, a uroplakin like Eg-TSP1 in *E. granulosus* was characterized and exhibited induction of a notable Th1 type immune response in a mouse model. Endogenous Eg-TSP1 was mainly expressed on the tegument of *E. granulosus* and was involved in maintenance of tegument structural integrity. These results suggest that Eg-TSP1 could be used as an intervention target for therapy, prevention and control of hydatid disease.

## Additional files

Additional file 1: Table S1. Real-time PCR primers involved in this study. Additional file 2: Table S2. Sequences of siRNAs. Additional file 3: Figure S1. Genome-wide phylogenetic analysis of *E. granulosus* tetraspanins. All tetraspanins from the genome of *E. granulosus* were employed to construct a phylogenetic tree using neighbor-joining method by MEGA software (version 5.05). The name of each tetraspanin is the accessing numbers of the gene in GeneDB (http://www.genedb.org/). The tetraspanins could be classified into 3 distinct branches represent the CD63 family, CD family and Uroplakin family, respectively. This phylogenetic tree was rooted by the Human-TSP1 (GenBank ID: NP_005718.2). Note: A total of 30 tetraspanin genes were reported in *E. granulosus* genome, but we can not find “EgrG_001021800.1” throughout the whole genome database.
