# Supplementary material for: Molecular insights into a tetraspanin in the hydatid tapeworm Echinococcus granulosus
Source: Parasit Vectors. 2015 Jun 10;8:311. doi: 10.1186/s13071-015-0926-y (PMC4464875; doi:10.1186/s13071-015-0926-y)
Supplement: Additional file 1: Table S1. — Real-time PCR primers involved in this study. [file 13071_2015_926_MOESM1_ESM.docx]

**Table S1. Real-time PCR primers involved in this study.**

| **Gene** | **Sense (5’- 3’)** | **Antisense (5’- 3’)** |
| --- | --- | --- |
| IL-12 | TGCTGGTGTCTCCACTCATGGC | TTTCAGTGGACCAAATTCCATT |
| IFN-γ | AACGCTACACACTGCATCTTGG | CAAGACTTCAAAGAGTCTGAGG |
| IL-4 | GAATGTACCAGGAGCCATATC | CTCAGTACTACGAGTAATCCA |
| IL-10 | CGGGAAGACAATAACTG | CATTTCCGATAAGGCTTG |
| HPRT | GTTGGATACAGGCCAGACTTTGTTG | GATTCAACTTGCGCTCATCTTAGGC |
| Eg-TSP1 | CGCCATTCCTGATAACCTAAA | TTCCCTTCGCATCCTACTACA |
| EF1α | TTTGAGAAAGAGGCGGCTGAGATG | TAATAAAGTCACGATGACCGGGCG |
