# Supplementary material for: Molecular insights into a tetraspanin in the hydatid tapeworm Echinococcus granulosus
Source: Parasit Vectors. 2015 Jun 10;8:311. doi: 10.1186/s13071-015-0926-y (PMC4464875; doi:10.1186/s13071-015-0926-y)
Supplement: Additional file 2: Table S2. — Sequences of siRNAs. [file 13071_2015_926_MOESM2_ESM.docx]

**Table S2. Sequences of siRNAs.**

| **siRNAs** | **Sense (5’-3’)** | **Antisense (5’-3’)** | **Targeted regions of EG-TSP1** |
| --- | --- | --- | --- |
| siRNA-132 | GGAUAAGGUCUCCUCCCAATT | UUGGGAGGAGACCUUAUCCTT | 132 – 150 bp |
| siRNA-480 | GCGUGGAUGACAACAAACUTT | AGUUUGUUGUCAUCCACGCTT | 480 – 498 bp |
| siRNA-540 | GCUACCGGGUCUUCAAUAUTT | AUAUUGAAGACCCGGUAGCTT | 540 – 558 bp |
| Control | UUCUCCGAACGUGUCACGUTT | ACGUGACACGUUCGGAGAATT | None |
