# Supplementary figures and images for: Molecular insights into a tetraspanin in the hydatid tapeworm Echinococcus granulosus
Source: Parasit Vectors. 2015 Jun 10;8:311. doi: 10.1186/s13071-015-0926-y (PMC4464875; doi:10.1186/s13071-015-0926-y)

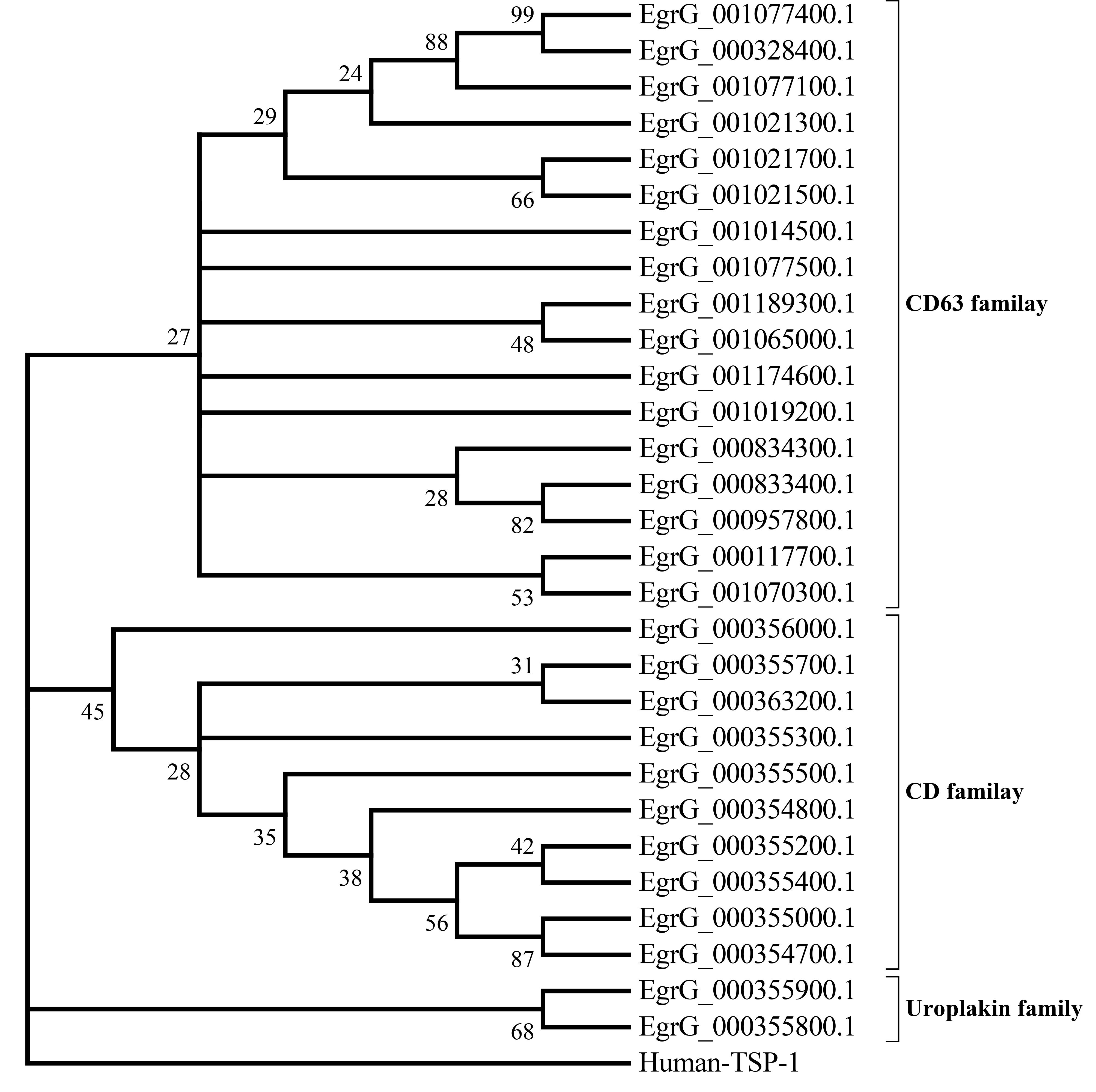

Supplement: Additional file 3: Figure S1. — Genome-wide phylogenetic analysis of E. granulosus tetraspanins. All tetraspanins from the genome of E. granulosus were employed to construct a phylogenetic tree using neighbor-joining method by MEGA software (version 5.05). The name of each tetraspanin is the accessing numbers of the gene in GeneDB (http://www.genedb.org/). The tetraspanins could be classified into 3 distinct branches represent the CD63 family, CD family and Uroplakin family, respectively. This phylogenetic tree was rooted by the Human-TSP1 (GenBank ID: NP_005718.2). Note: A total of 30 tetraspanin genes were reported in E. granulosus genome, but we can not find “EgrG_001021800.1” throughout the whole genome database. [file 13071_2015_926_MOESM3_ESM.tif]
